# Comparisons of Intravesical Treatments with Mitomycin C, Gemcitabine, and Docetaxel for Recurrence and Progression of Non-Muscle Invasive Bladder Cancer: Updated Systematic Review and Meta-Analysis

**DOI:** 10.3390/cancers16244125

**Published:** 2024-12-10

**Authors:** Jubin E. Matloubieh, David Hanelin, Ilir Agalliu

**Affiliations:** 1 Department of Urology, Montefiore Medical Center, Albert Einstein College of Medicine, Bronx, NY 10461, USA; jm5889@cumc.columbia.edu; 2 Albert Einstein College of Medicine, Bronx, NY 10461, USA; david.hanelin@einsteinmed.edu; 3 Department of Epidemiology and Population Health, Albert Einstein College of Medicine, Bronx, NY 10461, USA

**Keywords:** non-muscle-invasive bladder cancer (NMIBC), mitomycin C (MMC), gemcitabine (GEM), docetaxel (DOCE), recurrence, progression, systematic review, meta-analysis

## Abstract

**Background:** Non-muscle-invasive bladder cancer (NMIBC) comprises about 75% of all bladder cancers. Although NMIBC is treatable, it poses significant costs and burdens to patients due to high recurrence rates. We conducted an updated meta-analysis of studies that evaluated the efficacy of and outcomes after treatment with mitomycin C (MMC), gemcitabine (GEM), and docetaxel (DOCE) for NMIBC recurrence and progression. **Methods:** We searched the PubMed and Cochrane databases for observational cohort studies and randomized clinical trials (RCT) conducted between 2009 and 2022 that assessed the efficacy of GEM, DOCE, or MMC, alone or in combination, regarding NMIBC outcomes. A total of 49 studies that met the inclusion criteria were reviewed for their quality, sample size, outcomes, and potential for bias, and relevant data were extracted for the meta-analysis. Separate meta-analyses were performed to assess the risks of recurrence or progression when comparing GEM/DOCE or MMC vs. other treatments. Study heterogeneity was assessed by I^2^ statistics. **Results:** Among 31 studies comparing GEM or MMC to other treatments for NMIBC recurrence, there were statistically significant risk reductions of 24% for GEM (pooled relative risk (RR) of 0.76; 95% confidence interval (CI) 0.64–0.87) and 37% for MMC (pooled RR = 0.63; 95% CI 0.58–0.68). Recurrence-free survival (RFS) for GEM or MMC alone was 69.5% (95% CI 66.6–72.3%) and 67.2% (95% CI 66.2–68.2%), respectively. Studies assessing the combination of treatments had a pooled RFS of 44.6% (95% CI 40.4–48.7%). Fewer studies examined the risk of NMIBC progression, with large variability and inconclusive results across them. **Conclusions:** Our findings corroborate recent guidelines indicating that both GEM and MMC are effective treatments that reduce tumor recurrence and improve survival of NMIBC, although with large variability across the studies. Fewer studies evaluated DOCE treatment, with inconclusive results. Women and minorities were generally underrepresented, raising concerns about the generalizability of the findings and highlighting the importance of including a broader patient population in future RCTs.

## Simple Summary

We conducted an updated systematic review and meta-analysis of observational cohort studies and randomized clinical trials published between 2009 and 2022 that evaluated the efficacy of and outcomes after treatment with mitomycin C, gemcitabine, and docetaxel for non-muscle invasive bladder cancer recurrence and progression. Compared to other treatments, both gemcitabine and mitomycin C showed statistically significant risk reductions of 24% and 37% for tumor recurrence, respectively. Fewer studies examined the risk of progression, with large variability and inconclusive results. Our findings corroborate the current guidelines indicating that both gemcitabine and mitomycin C are effective treatments that reduce tumor recurrence and improve survival of non-muscle-invasive bladder cancer, although with large variability across studies. Given that women and minorities were generally underrepresented, our results highlight the importance of including a broader patient population in future clinical trials.

## 1. Introduction

Bladder cancer (BCa) is the sixth most diagnosed non-skin cancer in the United States and the ninth most common cancer worldwide, posing a significant disease burden worldwide [1,2]. The well-established risk factors for BCa are age, male sex, race and ethnicity, and tobacco smoking, as well as several environmental and occupational exposures [3,4,5]. Of the estimated 610,000 newly diagnosed BCa cases worldwide each year, about 75% of them are non-muscle-invasive bladder cancer (NMIBC), which is a genetically and clinically distinct entity from muscle-invasive BCa [2,6,7]. NMIBC is rarely lethal but has high rates of recurrence, leading to repeated tumor treatments of patients over extended periods of time [7]. Consequently, NMIBC places a relatively large social and economic burden on individuals and society, as each surveillance visit and treatment with repeated transurethral resection of the bladder tumor (TURBT) imposes significant time commitment, psychological distress, and monetary costs on patients [8,9,10]. Indeed, NMIBC is estimated to have the highest cost per patient from diagnosis to death among all cancers in the US Medicare system [8].

The main treatment modalities for NMIBC consist of a combination of TURBT, intravesical therapies, and occasionally systemic therapies. The earliest intravesical therapies that were effective for NMIBC treatment were the Bacillus Calmette–Guérin (BCG) immunotherapy and mitomycin C (MMC) [6,11,12]. Although these therapies remain the standard treatments for NMIBC, the most recent American and European Urological Association guidelines [13,14] recommend the risk-stratified use of intravesical therapeutics for NMIBC. These recommendations range from a single postoperative dose of intravesical chemotherapy with gemcitabine (GEM) or MMC after TURBT for low- or intermediate-risk NMIBC to induction followed by maintenance intravesical chemotherapy for intermediate-risk NMIBC. Further, intravesical chemotherapy is recommended for high-risk NMIBC after BCG failure when cystectomy is not an option for the patient. In addition to these treatments, new intravesical therapies such as docetaxel (DOCE) alone or in combination with GEM have been added to the treatment of high-risk NMIBC [6,13,14,15]. As patients fail therapy with intravesical BCG, or when there are BCG shortages, intravesical chemotherapeutics with either MMC, DOCE, or GEM (or a combination of treatments) become increasingly important in the treatment of intermediate- or high-risk NMIBC [13,14].

While intravesical chemotherapy for the treatment of NMIBC has been used for decades, few large randomized clinical trials (RCT) have evaluated the efficacy of these medications in relation to the risks of NMIBC recurrence or progression. Prior systematic reviews and a meta-analysis [16,17,18], which evaluated the effectiveness of intravesical treatment with GEM compared to either saline or MMC, suggested that GEM offered little benefit over saline for NMIBC recurrence. However, there was no benefit of GEM regarding either NMIBC progression or high-grade disease [16]. By contrast, when compared to MMC, the protective effect of GEM was more pronounced, with hazard ratios (HR) ranging from 0.36 to 0.57 for NMIBC recurrence [16]. Nevertheless, the certainty of the evidence from this systematic review was rather low, and there was no meta-analysis conducted to determine the summary effects of prior RCT studies.

Therefore, the goal of our study was to carry out a comprehensive systematic review and conduct a formal meta-analysis of the observational cohort studies and RCTs published since 2009, aiming to compare the efficacy of either GEM, DOCE, or MMC alone (or a combination of treatments) in relation to the risks of NMIBC outcomes: recurrence, high-grade (III to IV) disease persistence, and/or progression. We focused this meta-analysis on GEM, DOCE, and MMC as these medications are the most used treatments for NMIBC as a first-line therapy or after BCG installation failure.

## 2. Materials and Methods

### 2.1. Search Strategy and Selection Criteria for the Studies

**Figure 1:**
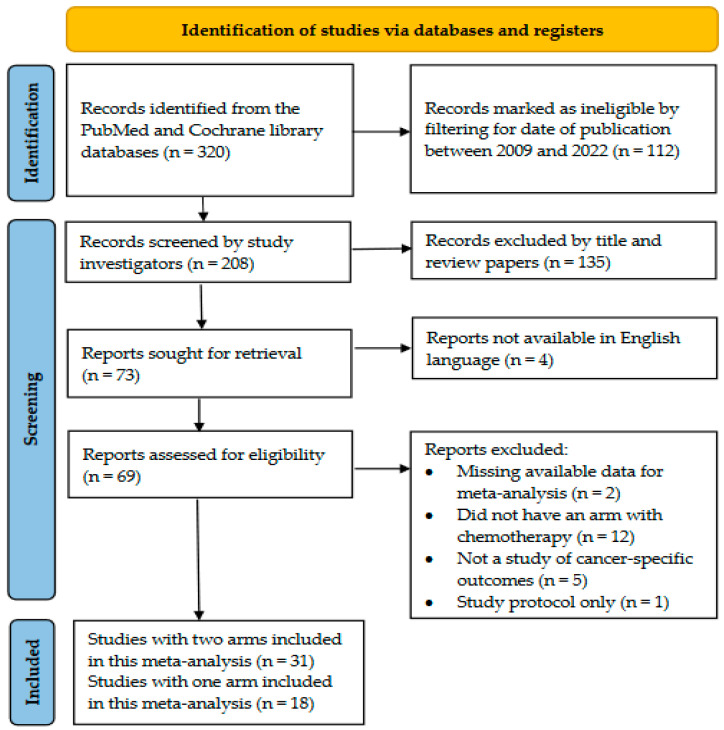
Flowchart of study selection for this updated systematic review and meta-analysis.

A literature search of the PubMed and Cochrane Library databases was performed by one of the investigators (JEM) using the following query terms: “intravesical AND gemcitabine OR mitomycin C, OR docetaxel” AND “non-muscle invasive” AND “bladder cancer” OR “urothelial carcinoma”. Moreover, the publication dates of studies were restricted to between 1 January 2009 and 31 December 2022. The exclusion criteria for studies were as follows: review articles for NMIBC with no detailed information on specific study treatments, sample size, or outcomes of interest; articles not available in the English language; laboratory studies conducted on animal models or cell lines; case reports; clinical studies that did not include any intravesical chemotherapy alone or in combination; clinical studies where the treatment for NMIBC was hyperthermic or electromotive therapy; and studies focusing only on the quality of life after NMIBC diagnosis, without reporting results regarding cancer-specific outcomes. The inclusion criteria were either observational retrospective (RC) or prospective cohort studies (PC) or RCTs that compared the efficacy of intravesical treatment with either MMC, GEM, or DOCE alone or their combination (e.g., GEM/DOCE or GEM/MMC) to other treatments (e.g., saline, BCG, epirubicin, pirarubicin, and interferon gamma (IFN), without the use of electromotive or hyperthermic therapies), in relation to high-grade tumors or the recurrence or progression of NMIBC. The PROSPERO registration number of this systematic review and meta-analysis is CRD42023400694. Figure 1 shows the flowchart used to select the studies included in this meta-analysis based on the inclusion and exclusion criteria. Given the paucity of clinical observational cohort studies or RCTs on this topic, particularly for GEM and/or DOCE studies, we also reviewed and included studies that were published in a recent systematic review and a prior meta-analysis [16,17].

### 2.2. Data Extraction and Statistical Data Analysis

All studies that met the inclusion criteria (Figure 1) were carefully evaluated by two investigators (JEM and IA), and all relevant data and information needed for the systematic review and meta-analysis were extracted (Table 1 and Table 2). This included the first author, year of publication, country where the study was conducted, study design (e.g., RC, PC, or RCT), sample size, patient characteristics (e.g., average age, sex, race, and ethnicity distribution), treatment arm (e.g., GEM, MMC, DOCE, or combination of treatments), and comparison group (e.g., saline, BCG, epirubicin, pirarubicin, IFN, etc.), with the corresponding number of patients included in each arm, outcomes of interest (e.g., high-grade tumor persistence, recurrence, recurrence-free survival, and progression), number of events for each treatment and comparison arm, and study results, i.e., relative risks (RR) and their corresponding 95% confidence intervals (CIs). If studies did not report the RR and 95% CI, then the RR and the corresponding 95% CI were computed by comparing the rates of recurrence in the treatment arm (e.g., GEM or MMC) to the rates of recurrence in the comparison group to determine the strength of the association of these treatments with the risk of recurrence, progression, or high-grade cancer. Studies included in the meta-analysis were also evaluated for quality using the Cochrane risk of bias tool [19], which assessed the study quality, sample size, and potential biases in the design and analysis, as well as confounding. Based on these criteria, a qualitative score of either poor, moderate, good, or very good was given to each study.

After data extraction, several meta-analyses were performed. We carried out separate meta-analyses and generated forest plots of the studies comparing either GEM or MMC alone (or any combination therapy) with standard therapy (e.g., BCG) or other treatments for NMIBC (e.g., saline, INF gamma, or other chemo agents) in relation to the risks of recurrence or progression by using the “metan” command in STATA [20]. The heterogeneity across studies was assessed by the I^2^ statistic [20], with I^2^ > 50% indicating statistically significant heterogeneity across studies. Due to the large statistical heterogeneity among the studies, we used a random-effects model throughout various meta-analyses. A statistical test with a two-sided *p* < 0.05 was considered statistically significant. We also carried out stratified meta-analyses by study design (i.e., RCT vs. observational cohorts). Sensitivity analyses were performed to examine whether the findings in the various meta-analyses were robust by excluding the most influential studies. Publication bias was also investigated by the Begg funnel plot trim-and-fill method, as well as the Egger regression test [21,22], with an asymmetric plot suggesting possible publication bias.

For single-arm studies, we also extracted the proportion of patients with recurrence-free survival (RFS) and their corresponding 95% CI at 24 months after treatment for NMIBC (if reported by specific studies) or at the end of follow-up for either the GEM, DOCE, or MMC arm alone or their combination. If RFS was not reported, we used data from various tables and figures in specific studies to estimate the RFS at 24 months of follow-up after treatment. Then, the corresponding 95% CI was calculated for the proportion of patients without recurrence using the binomial “exact” calculation method [23]. We carried out separate meta-analyses and generated forest plots of the RFS for observational cohort studies or RCTs that used either GEM alone, MMC alone, or any combination therapy (e.g., GEM/DOCE or GEM/MMC), using the “metan” command as described above. All statistical analyses were performed by using STATA version 18 (Stata Corporation, College Station, TX, USA).

## 3. Results

A total of 320 records were identified from the PubMed and Cochrane databases, of which 271 were deemed ineligible based on the screening and filtering criteria, as shown in the PRISMA flowchart in Figure 1. A total of 49 studies were eligible for our review, of which 31 studies were included in the main meta-analysis that compared GEM, DOCE, and/or MMC to other treatments in relation to the risk of NMIBC recurrence, respectively (Table 1 and Table 2). An additional 18 single-arm studies, which were not eligible for inclusion in the main meta-analysis because they lacked a comparison arm, were included in the meta-analyses of either GEM or MMC that investigated RFS in NMIBC. The studies included in these meta-analyses are listed in Table 1 and Table 2, along with a summary of their characteristics and assessments of the study quality. The quality of the GEM vs. other treatment studies ranged from fair to very good, and the rating of the evidence ranged from moderate to high (Table 1). The quality of the studies that compared MMC to other treatments ranged from good to very good, and the evidence ranged from poor to high (Table 2). The quality of the single-arm studies ranged from poor to very good; however, we did not assess their evidence due to the lack of a comparison group.

### 3.1. GEM and/or DOCE Treatment and Risks of NMIBC Recurrence or Progression

Thirteen studies [24,25,26,27,28,29,30,31,32,33,34,35] that compared GEM and/or DOCE to other treatments in relation to the risk of NMIBC recurrence, with a total number of 1989 patients (950 in the treatment arm and 1039 in the control arm), were included in the main meta-analysis (Table 1). Eight of the studies (61.5%) were RCTs [24,25,26,27,28,30,32,36], and the remaining five studies (38.5%) were RC studies. The average age of the NMIBC patients across these 13 studies was 67.7 years old, and most patients were male (80%). There were variations across the studies regarding the treatment types. Eleven studies [24,26,27,28,29,30,31,32,33,34,36] compared the effect of GEM alone to other treatments, which varied across studies, including BCG, saline, placebo, MMC, epirubicin, or pirarubicin, in relation to the risk of recurrence and/or progression (Table 1). The remaining two studies compared either a combination of GEM and BCG treatment to BCG alone [25] or GEM/DOCE to BCG and IFN treatment (control arm) [35] in relation to the risk of NMIBC recurrence. Overall, the quality of the “GEM vs. other treatment studies” ranged from fair to very good, and the quality of the evidence ranged from moderate to high (Table 1).

**Figure 2:**
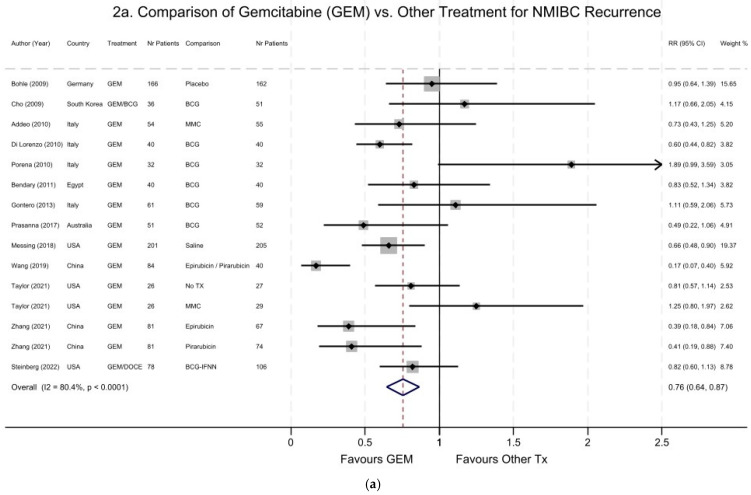

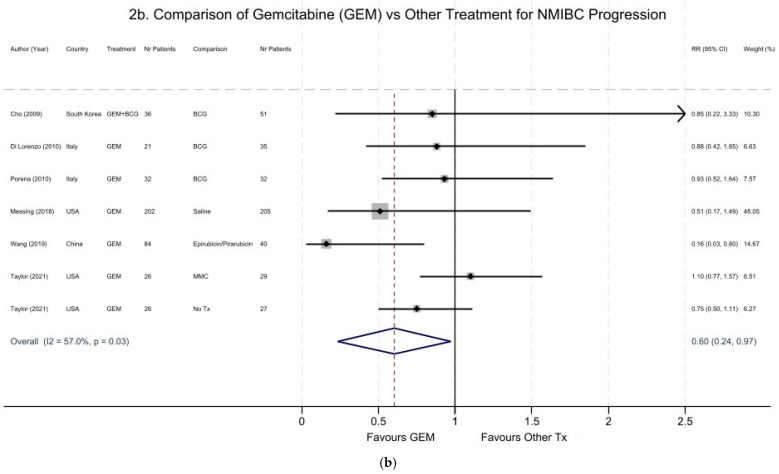
Forest plot of studies comparing the efficacy of gemcitabine (GEM) and/or docetaxel (DOCE) vs. other treatments in relation to the risks of recurrence (**a**) and of high-grade disease persistence and progression (**b**) for non-muscle-invasive bladder cancer (NMIBC). (**a**) NMIBC recurrence. Summary pooled RR = 0.76 (95% CI 0.64–0.87)—favors gemcitabine (GEM). Overall heterogeneity: I^2^ = 80.4% (*p* < 0.0001). Included studies comparing GEM alone (*n*=13) or a combination of GEM/BCG (*n* = 1) or GEM/DOCE (*n* = 1) to other treatments. Studies were weighted by their sample size; studies with a larger number of patients, indicated by larger grey boxes, received greater weights. (**b**) High-grade NMIBC persistence and progression. Summary pooled RR = 0.60 (95% CI 0.22–0.97)—favors GEM. Overall heterogeneity: I^2^ = 57% (*p* = 0.03). Included studies comparing GEM or GEM/BCG to other treatments that reported risks of progression or high-grade disease persistence. Bohle et al.’s RCT study [24] was removed from this analysis due to an unstable RR of 3.0 with a very large 95% CI (0.32–28.45).

Figure 2 shows the forest plot of studies comparing the efficacy of GEM and/or DOCE vs. other treatments in relation to the risks of recurrence (Figure 2a) and progression (Figure 2b) for NMIBC. Overall, there was a 24% reduction in the risk of NMIBC recurrence associated with GEM and/or DOCE treatment, with a summary pooled RR of 0.76 (95% CI 0.64–0.87; Figure 2a), compared to all other treatments. However, there was large heterogeneity across the studies, with an overall I^2^ = 80% (*p* < 0.0001). When we restricted the meta-analysis to the 11 studies that compared GEM only vs. other treatments, the summary RR was 0.73 for NMIBC recurrence (95% CI 0.61–0.85). Finally, when we stratified the analyses by study design, the reduction in the risk of NMIBC recurrence associated with the GEM treatment was more pronounced in observational studies (RR = 0.52; 95% CI 0.38–0.65) compared to RCTs (pooled RR = 0.90; 95% CI 0.74–1.06; Supplementary Figure S1).

Seven studies [24,25,27,28,32,33,36] reported data on NMIBC high-grade persistence or progression. However, an RCT study [24] conducted in Germany was excluded from this analysis due to the small number of NMIBC progression events, with an unstable RR estimate and a very large 95% CI (RR = 3.0; 95% CI 0.32–28.45). Overall, there was an inverse association between GEM treatment and the risk of NMIBC progression (pooled RR = 0.60; 95% 0.22–0.97); however, there was large variability and heterogeneity across the studies (Figure 2b). The RCT conducted in the US by Messing and colleagues [32] was the largest study and thus carried the highest weight in this meta-analysis.

We also examined the potential for publication bias in RCTs and observational cohorts that examined the effect of GEM alone or GEM/DOCE treatments on the risk of NMIBC recurrence. The funnel plot showed suggestive evidence of publication bias, with four potential missing studies on the left side of the plot (imputed or missing studies are indicated with yellow dots in Supplementary Figure S2).

### 3.2. MMC Treatment and Risks of NMIBC Recurrence or Progression

A total of 18 studies [37,38,39,40,41,42,43,44,45,46,47,48,49,50,51,52,53,54] that compared MMC to other treatments in relation to the risk of NMIBC recurrence, with a cumulative number of 7159 patients (4054 in the treatment arm and 3108 in control arm), were included in the main meta-analysis. Of these, the multicenter RCT study conducted by Xylinas et al. [49] contributed 59% of all patients (Table 2). Most of the studies (72%) were RCTs, with five studies being retrospective cohorts [38,41,45,50,52]. The average age of the NMIBC patients across the studies was 67.9 years, and the majority (79%) were male; however, there was some variation across the studies. Most studies (*n* = 13) compared the effect of MMC alone to other treatments, which varied across studies, including BCG, transurethral resection (TUR), placebo, epirubicin, or pirarubicin, in relation to the risk of recurrence and/or progression (Table 2). A few studies compared the MMC and BCG or TUR combination to other treatments [40,43,44,48,53]. Overall, the quality of the studies included in the final meta-analysis ranged from moderate to high (Table 2). We excluded a study conducted in Belgium [45] due to its very small number of patients in the control arm (*n* = 3) with no recurrence events.

**Figure 3:**
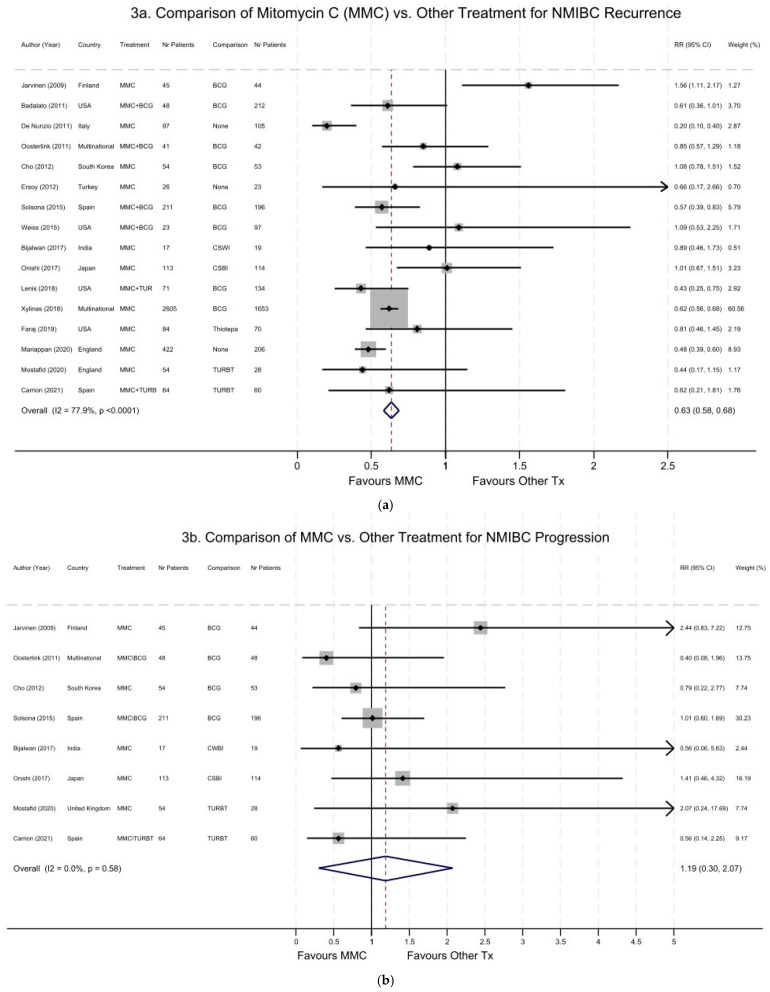
Forest plot of studies comparing the efficacy of mitomycin C (MMC) vs. other treatments in relation to the risks of recurrence (**a**) and of high-grade disease persistence and progression (**b**) for non-muscle-invasive bladder cancer (NMIBC). (**a**) NMIBC recurrence. Summary pooled RR = 0.63 (95% CI 0.58–0.68)—favors MMC. Overall heterogeneity: I^2^ = 78% (*p* < 0.0001). Studies included comparing MMC alone or MMC and BCG therapy to other treatments. Studies with a larger sample size (i.e., number of patients), as indicated by larger grey boxes, received greater weight. This meta-analysis was highly influenced by a large study [49] that contributed 61% of all weight. (**b**) High-grade NMIBC persistence and progression: summary pooled RR = 1.19 (95% CI 0.30–2.07). Overall heterogeneity: I^2^ = 0% (*p* = 0.58).

Figure 3 shows the forest plot of the studies comparing the efficacy of MMC vs. other treatments in relation to the risks of NMIBC recurrence (Figure 3a) and progression (Figure 3b). Overall, there was a 37% reduction in the risk of NMIBC recurrence associated with MMC treatment, with a summary pooled RR of 0.63 (95% CI 0.58–0.68; Figure 3a), compared to all other treatments. However, there was large heterogeneity across the studies, with an overall I^2^ = 78% (*p* < 0.0001). This meta-analysis was highly influenced by a large RCT study [49], which contributed 60.6% of the weight of all included studies. When we excluded this study and redid the meta-analysis, the summary RR for NMIBC recurrence did not drastically change (pooled RR = 0.65; 95% CI 0.56–0.75). When the analysis was stratified by study design, the summary pooled RR for NMIBC recurrence was similar between RCTs versus observational cohorts (Supplementary Figure S3). However, there was no association between MMC treatment and the risk of high-grade disease persistence or NMIBC progression (RR = 1.19, 95% CI 0.30–2.07, Figure 3b). There was also a higher likelihood of publication bias, with many missing or imputed studies, as indicated by the yellow dots in Supplementary Figure S4.

### 3.3. Recurrence-Free Survival (RFS) for NMIBC in Relation to GEM, MMC, or the Combination of Treatments

**Figure 4:**
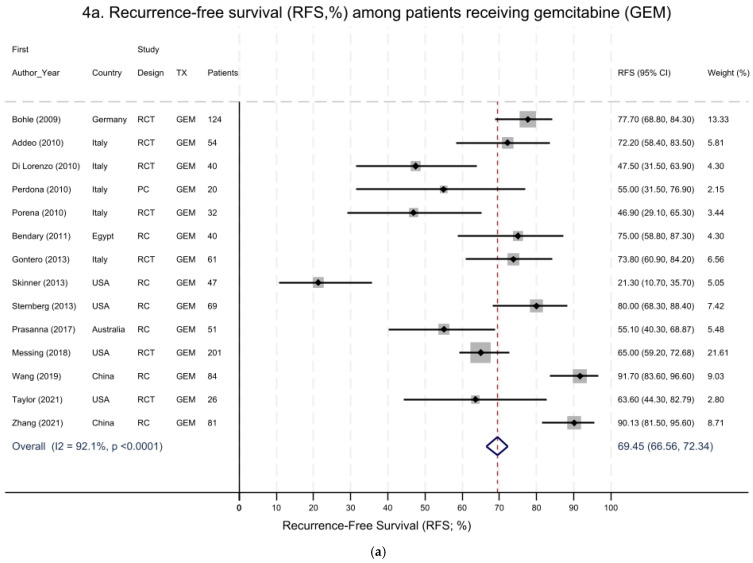

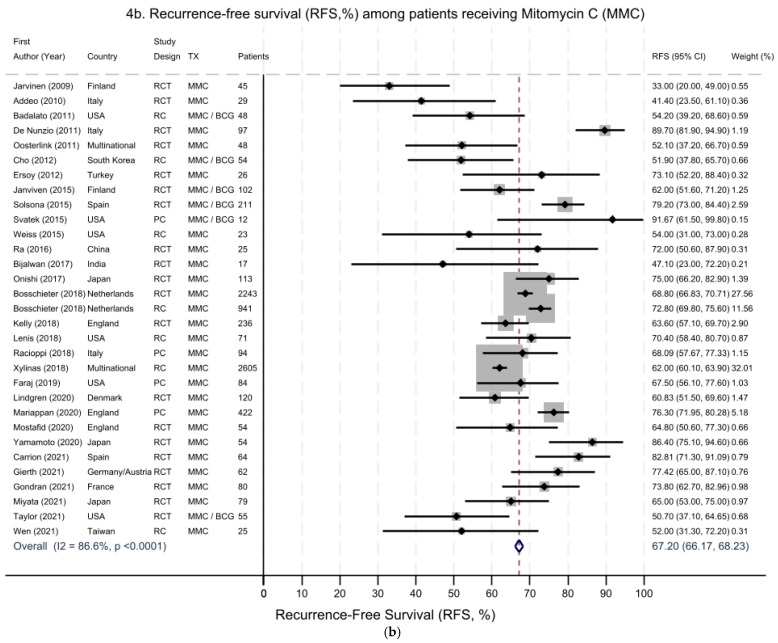

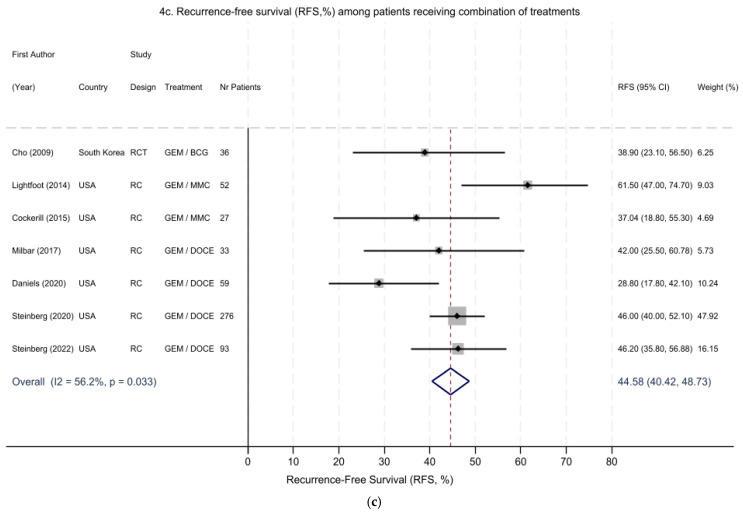
Recurrence-free survival (RFS) during follow-up of NMIBC patients receiving either gemcitabine (GEM) alone (**a**), mitomycin C (MMC) alone (**b**), or the combination of treatments (**c**). (**a**) GEM treatment: summary pooled RFS = 69.5% (95% CI 66.6–72.3%). Overall heterogeneity: I^2^ = 92% (*p* < 0.0001). Studies included evaluating RFS among patients receiving GEM treatment only. Studies with a larger number of patients, as indicated by larger grey boxes, received greater weights. (**b**) MMC treatment: summary pooled RFS = 67.2% (95% CI 66.2–68.2%). Overall heterogeneity: I^2^ = 86.6% (*p* < 0.0001). Studies included evaluating RFS among patients receiving MMC alone or MMC/BCG treatment. (**c**) Combination of treatments: summary pooled RFS = 44.6% (95% CI 40.4–48.7%). Overall heterogeneity: I^2^ = 56% (*p* = 0.033). Studies included evaluating RFS among NMIBC patients receiving various combinations of treatments (e.g., GEM/BCG, GEM/MMC or GEM/DOCE). Studies with a larger number of patients, as indicated by larger grey boxes, received greater weights.

We also performed separate meta-analyses of recurrence-free survival (RFS) for NMIBC in relation to GEM-only treatment (Figure 4a), MMC-only (Figure 4b), or combinations of treatments (i.e., GEM and DOCE, or MMC; Figure 4c) using the two-arm studies reported above, as well as the single-arm studies of GEM or MMC alone or in combination (Table 1 and Table 2) [55,56,57,58,59,60,61,62,63,64,65,66,67,68,69,70,71,72,73]. Regarding GEM-only treatment, there was a pooled RFS of 69.5% (95% CI 66.6–72.3%) for NMIBC (Figure 4a); however, there was large heterogeneity among the 14 studies (I^2^ = 92%, *p* < 0.0001). Similar findings were also observed for MMC treatment alone, with a pooled RFS of 67.2% (95% CI 66.2–68.2%) for NMIBC (Figure 4b); again, there was large heterogeneity across these 31 studies, with an overall I^2^ = 86.6% (*p* < 0.0001). Two studies in the MMC group with the largest sample size had the highest weights of 27% and 32%, respectively [49,67]. When we excluded these two influential studies, the pooled RFS for NMIBC was 70.2% (95% CI 68.6–71.8%). The combination of GEM with other treatments, including DOCE (four studies), MMC (two studies), or BCG (one study), showed a pooled RFS of 44.6% (95% CI 40.4–48.7%) for NMIBC (Figure 4c). Again, there was significant heterogeneity across the studies (I^2^ = 56%; *p* = 0.033).

## 4. Discussion

In this updated meta-analysis, we included a wide range of RCTs and observational cohort studies that evaluated the effects of GEM, MMC, or a combination of treatments (i.e., GEM/DOCE; GEM/MMC) in relation to the risk of NMIBC recurrence, as well as disease persistence or tumor progression. Our results showed that there were statistically significant 24% and 37% reductions in the risk of NMIBC recurrence associated with GEM and MMC treatment, respectively. By contrast, there was less clear evidence of the benefit of these treatments in relation to NMIBC persistence or progression. Although there was an inverse association between GEM treatment and the risk of NMIBC progression (pooled RR = 0.60; 95% CI 0.22–0.97), there was large variability across the studies, with most of them showing no clear association. Of note, there was large statistical heterogeneity across the RCTs and observational cohort studies, as well as a wide range of rating regarding the study quality and quality of evidence across the different studies included in the various meta-analyses. Finally, the dose, duration, and schedule of the intravesical treatments; the comparison groups; and tumor grade and stage varied substantially across the studies. Taken together, these factors could explain the large variation and differences in the results.

A few prior systematic reviews and meta-analyses [16,17,18] were carried out to evaluate the effectiveness of the intravesical instillation of GEM alone compared to either saline or MMC in relation to NMIBC outcomes, with inconsistent results. Han et al. used data from seven RCTs and reported that intravesical GEM treatment was associated a 33% reduction in the risk of NMIBC recurrence compared to saline; however, the results were not statistically significant (RR = 0.77; 95% CI 0.54–1.09) [16]. When compared to MMC, another meta-analysis reported that the protective effect of GEM treatment was more pronounced, with an RR of 0.44 (95% CI 0.24–0.78) for NMIBC recurrence [18]. There was, however, no benefit of GEM treatment regarding either NMIBC progression or high-grade adverse events. It should be noted that most of the studies included in these prior meta-analyses had a limited number of patients and recurrence events in both the treatment and comparisons groups; therefore, they had limited statistical power to observe a modest effect.

In our meta-analysis, the studies evaluating the effects of MMC vs. other treatments were consistently of higher quality and contained more high-quality evidence compared to the other meta-analysis. A greater proportion were RCTs, and one observational cohort study had enrolled a large number of patients [52]. Only one study was deemed to have poor-quality evidence [45], but its effect on the meta-analysis was minimal as it was by far the smallest study. Almost all the cohorts compared MMC to placebo; only 3.5% of the studies compared MMC to another chemotherapy or included BCG as part of the intravesical cocktail treatment. The overall effect of MMC was positive in terms of reducing the risk of NMIBC recurrence, a finding that has been corroborated in a prior meta-analysis [74], cementing its place as a primary chemotherapeutic for NMIBC. A plausible biological explanation for the observed differences between MMC vs. GEM would be the higher logarithmic partition coefficient of MMC, which could positively affect the intravesical absorption and tumor infiltration of MMC compared to GEM [75].

By contrast, the studies evaluating GEM’s efficacy vs. other treatments were of mixed quality. Although most of them were RCTs (64%), the comparison group varied substantially and included a variety of treatments, such as BCG, placebo, MMC, epirubicin, pirarubicin, or a combination of treatments. Therefore, this heterogeneity could explain in part the differences that we observed between the RCTs and observational cohort studies regarding the reduction in the risk of NMIBC recurrence associated with GEM treatment. There was also significant variability among the studies in the dose, duration, and frequency of intravesical treatments, and therefore any observed differences in the results could be due to these conditions. Moreover, observational studies are prone to selection bias regarding treatment and confounding by indication, which could affect their results. When the meta-analyses were restricted to 11 studies that compared GEM alone to other treatments, the pooled RR was 0.73 (95% CI 0.61–0.85) for NMIBC recurrence. Although MMC had a pooled RR of 0.63 (95% CI 0.58–0.68) for NMIBC recurrence, the 95% CIs for the two meta-analyses overlapped. Therefore, the difference in risks of tumor recurrence between GEM and MMC treatments was not statistically significant. In relation to NMIBC progression, overall, fewer studies evaluated this outcome, with a smaller number of events and inconclusive results.

Finally, the pooled single-arm meta-analyses were also derived from studies whose quality ranged from poor to very good, and they contained a mixture of RCTs and observational cohort studies. Again, the treatments varied across these studies and included single chemotherapeutics (e.g., GEM, DOCE, or MMC) as well as combinations thereof (GEM/DOCE, GEM/MMC, and MMC/BCG). Despite the heterogeneity, GEM treatment alone had the strongest effect in terms of increasing RFS, followed by MMC alone, GEM+MMC, and GEM+DOCE. The difference in the RFS effect between GEM and MMC was modest and based on a larger number of studies and patients receiving MMC compared to GEM alone. In addition, the meta-analysis of the RFS included several other single-arm studies that did not have a comparison group to be included in the main meta-analysis (Table 1 and Table 2). Therefore, the results might not be directly comparable between the different meta-analyses shown in Figure 2, Figure 3 and Figure 4. If more patients were included in studies that received GEM treatment, we anticipate that we would have observed an RFS treatment effect that more closely resembled that of MMC.

Our study has several strengths. This was a large meta-analysis, which included both RCTs and observational cohort studies that compared the effects of GEM or MMC in relation to all other treatments. Our meta-analyses included many patients, both in the treatment and control arms, and thus the analyses were well powered to detect both moderate and modest effect sizes. In addition, we also included single-arm studies to determine the RFS associated with either GEM or MMC treatment alone (or a combination of treatments) to increase the precision of our estimates. We chose to include RCTs and observational cohort studies, as well as single-arm studies, to maximize the information on the treatment effects that could be observed in various situations. However, observational studies are prone to confounding by indication or selection bias regarding treatment, which could potentially affect their results and therefore explain the difference obtained from RCTs.

There are several other limitations that should be carefully considered when evaluating the evidence. First, there was large heterogeneity across the studies in their design; the dose, duration, and schedule of the intravesical treatments; the comparison control group; the tumor grade and stage; and the effect size estimates and their corresponding 95% CIs. Therefore, some of the results could be partly explained by this large heterogeneity. Despite these differences, our sensitivity analyses showed that, in general, the results were robust and were less affected by single study results or outliers. Moreover, there was large variability in the follow-up of patients for recurrence or progression; although the largest studies included in our meta-analyses followed-up their NMIBC patients for at least three years or longer. Notably, most studies were carried out in Western countries (e.g., US and Europe) and East Asia, and no studies were conducted in Latin America, Sub-Saharan Africa, South/Southeast Asia, or Eastern Europe. Therefore, some of the findings might not be generalizable to different patient populations. Finally, women comprised less than 25% of all participants, and few studies reported on patients’ races and ethnicities, perhaps limiting the generalizability of the results. Although the sex and racial distribution is consistent with the demographic characteristics of NMIBC patients [2,3,7], other studies have shown that women are more likely to be diagnosed with higher-stage NMIBC and they have higher risks of recurrence, progression, and cancer-specific mortality [76]. Therefore, more representative cohorts of patients need to be enrolled and evaluated in large RCTs to determine whether the results would be similar across genders or different populations.

## 5. Conclusions

This large systematic review and meta-analysis showed that both GEM and MMC are effective treatments that reduce tumor recurrence and improve survival for NMIBC patients, although with large variability across studies. These results corroborate the updated guidelines for NMIBC treatment from both the American and European Urological Associations [13,14]. Few studies have evaluated DOCE, with inconclusive results; therefore, further research is needed to determine its efficacy. Women and minorities were generally underrepresented in these studies, highlighting the importance of including a broader patient population in future RCTs to further understand how these chemotherapeutic agents will benefit various patients.

## Figures and Tables

**Table 1:** Characteristics of studies included in the meta-analysis of gemcitabine (GEM) and/or docetaxel (DOCE) for the treatment of non-muscle-invasive bladder cancer (NMIBC).

First Author	Year	Country	Study Design	Age	Men (%)	Treatment Arm	Nr Pts	Control Arm	Nr Pts	Follow-Up (m)	Study Quality/ Evidence	Outcomes Studied
**Gemcitabine (GEM) vs. Other Treatment**												
Bohle	2009	Germany	RCT	66	80%	GEM	166	PBO	162	38.6	Very Good/ High	RR. RFS
Cho	2009	South Korea	RCT	63	92%	GEM/BCG	36	BCG	51	32.2	Very Good/ Moderate	RR, RFS Prog
Addeo	2010	Italy	RCT	66	85%	GEM	54	BCG	55	36	Good/ Moderate	RR, RFS
Porena	2010	Italy	RCT	70	84%	GEM	32	BCG	32	44	Good/ Moderate	RR, HGRR
DiLorenzo	2010	Italy	RCT	70	61%	GEM	40	BCG	40	12	Good/ Moderate	RR, RFS Prog
Bendary	2011	Egypt	RCo	56	NR	GEM	40	BCG	40	10.8	Fair/Moderate	RR
Gontero	2013	Italy	RCT	67	86%	GEM	61	BCG	59	10.5	Very Good/ Moderate	RR
Prasanna	2017	Australia	RCo	78	84%	GEM	51	BCG	52	15	Fair/Moderate	RFS
Messing	2018	USA	RCT	66	85%	GEM	201	Saline	205	48	Very Good/ High	RR, Prog, OS, HGRR
Wang	2019	China	RCo	NR	73%	GEM	84	Epi	40	35.2	Good/ Moderate	RR Prog RFS OS
Zhang	2021	China	RCo	63	70%	GEM	81	Epi	67	24	Good/ Moderate	RR
								Pira	74			
Taylor	2021	USA	RCT	73	82%	GEM	26	PBO	27	25.2	Good/High	RFS, HGRR
								MMC	29			
Steinberg	2022	USA	RCo	74	78%	GEM/DOCE	78	BCG/IFN	106	24	Very Good/ Moderate	RFS, HGRR
**Single-Arm Studies Involving Gemcitabine (GEM) and/or Docetaxel (DOCE) Included for Cancer-Specific Outcomes**												
Perdonà	2010	Italy	PC	68	65%	GEM	20			15	Good/NA	RR, RFS, Prog
Skinner	2013	USA	PC	70	79%	GEM	47			24	Very Good/NA	RR, RFS, Prog
Sternberg	2013	USA	RCo	72	77%	GEM	69			42	Very Good/NA	OS, RFS, Prog
Cockerill	2016	USA	RCo	74	89%	GEM/MMC	27			22	Fair/NA	RR
Milbar	2017	USA	RCo	73	70%	GEM/DOCE	33			18.6	Poor/NA	RFS, HGRR
Shantharam	2021	USA	RCo	75	46%	DOCE	13			12	Poor/NA	RR

BCG = Bacille Calmette–Guérin, DFS = Disease-Free Survival, Epi = Epirubicin, GEM = Gemcitabine, HGRR = High-Grade Disease Recurrence, IFN = Interferon, MMC = Mitomycin C, NA = Not Applicable, NR = Not Reported, OS = Overall Survival, PBO = Placebo, PC = Prospective Cohort, Pira = Pirarubicin, Prog = Progression, RCo = Retrospective Cohort, RCT = Randomized Controlled Trial, RFS = Recurrence-Free Survival, RR = Recurrence Rate, TUR = Transurethral Resection. Evidence quality is not applicable (NA) for single-arm studies.

**Table 2:** Characteristics of studies included in the meta-analysis of mitomycin C (MMC) for the treatment of non-muscle-invasive bladder cancer (NMIBC).

First Author	Year	Country	Study Design	Age	Men (%)	Treatment Arm	Nr Pts	Control Arm	Nr Pts	Follow-Up (m)	Study Quality Evidence	Outcomes Studied
**Mitomycin C (MMC) vs. Other Treatment**												
Jarvinen	2009	Finland	RCT	67	72%	MMC	45	BCG	44	102	Very Good/ High	OS, DFS, Prog
Badalato	2011	USA	RCo	70	71%	MMC	48	MMC/BCG	212	34.5	Very Good/ Moderate	RFS
De Nunzio	2011	Italy	RCT	61	63%	MMC	97	TUR	105	90	Very Good/ High	RFS, RR
Oosterlinck	2011	Multinational	RCT	69	83%	MMC/BCG	41	BCG	42	56.4	Very Good/ High	CR, Prog, DFS, RR
Cho	2012	South Korea	RCo	64	81%	MMC	54	BCG	53	24.3	Good/Moderate	RFS, RR, Prog
Ersoy	2013	Turkey	RCT	61	90%	MMC	26	TUR	23	60	Good/Moderate	RFS
Solsona	2015	Spain	RCT	67	90%	MMC/BCG	211	BCG	196	85.2	Very Good/High	RR, Prog, OS, RFS
Weiss	2015	USA	RCT	64	75%	MMC/BCG	23	BCG	97	54	Good/Moderate	RFS, OS
Decaestecker	2016	Belgium	PC	70	88%	MMC	25	Epi	3	35	Good/Poor	CR, RFS
Bijalwan	2017	India	RCT	65	92%	MMC	17	CBI	19	12	Very Good/ Moderate	RFS, RR, Prog
Onishi	2017	Japan	RCT	72	80%	MMC	113	CBI	114	37	Very Good/ High	RFS
Lenis	2018	USA	RCT	72	81%	MMC/TUR	71	TUR	134	48	Very Good/ High	RFS
Xylinas	2018	Multinational	RCT	66	79%	MMC	2605	PBO	1653	60	Very Good/ High	RFS
Faraj	2019	USA	RCo	76	75%	MMC	84	Thiotepa	70	20	Good/Moderate	RR, RFS
Mostafid	2020	England	RCT	72	77%	MMC	54	TUR	28	24	Good/Moderate	CR, RFS
Mariappan	2020	England	PC	72	71%	MMC	422	PBO	206	63	Good/High	RR
Yamamoto	2020	Japan	RCT	65	74%	MMC/TUR	54	TUR/Pira	49	33	Good/Moderate	RFS, RR
Carrión	2021	Spain	RCT	70	75%	MMC	64	PBO	60	15	Very Good/ High	RR, DP, RFS
**Single-Arm Studies Involving Mitomycin C (MMC) Included for Cancer-Specific Outcomes**												
Colombo	2012	Italy	RCo	63	78%	MMC	54			NR	Moderate/NA	RFS, RR, Prog
Svatek	2015	USA	PC	67	58%	MMC/BCG	12			60	Poor/NA	RFS
Jarvinen	2015	Finland	RCT	68	72%	MMC/BCG	102			120	Good/NA	RR, Prog, RFS, OS
Ba	2017	China	RCT	52	92%	MMC	25			47	Good/NA	RR, RFS
Bosscheiter	2018	Netherlands	RCo	67	81%	MMC	941			34	Very Good/NA	RFS
Bosscheiter	2018	Netherlands	RCT	68	82%	MMC	2243			51	Very Good/NA	RR, RFS
Kelly	2019	England	RCT	67	79%	MMC	236			58	Very Good/NA	RR
Racioppi	2019	Italy	PC	65	85%	MMC	94			39	Fair/NA	CR, RFS
Lindgren	2020	Denmark	RCT	71	72%	MMC	129			3	Good/NA	CR, RFS
Miyata	2022	Japan	RCT	73	79%	MMC	79			62	Good/NA	RFS
Gierth	2021	Germany/Austria	RCT	69	78%	MMC	62			22	Good/NA	RFS
Wen	2021	Taiwan	RCo	75	74%	MMC	25			24	Fair/NA	RR
Gondran	2021	France	RCo	71	80%	MMC	167			12	Fair/NA	RFS

BCG = Bacille Calmette–Guérin, CBI = Saline Continuous Bladder Irrigation, CR = Complete Response, DFS = Disease-Free Survival, Epi = Epirubicin, MMC = Mitomycin C, NA = Not Applicable, NR = Not Reported, OS = Overall Survival, PBO = Placebo, PC = Prospective Cohort, Pira = Pirarubicin, Prog = Progression, RCo = Retrospective Cohort, RCT = Randomized Controlled Trial, RFS = Recurrence-Free Survival, RR = Recurrence Rate, TUR = Transurethral Resection, TTR = Time to Recurrence. Evidence quality not applicable (NA) to single-arm studies.

## Data Availability

The data used for the systematic review and meta-analyses are available upon request from the corresponding author. All relevant studies included in the meta-analyses are already published and data are available through PubMed.

## Supplementary Materials

The following supporting information can be downloaded at: https://www.mdpi.com/article/10.3390/cancers16244125/s1, Figure S1: Forest plot of studies comparing the efficacy of gemcitabine (GEM) and/or docetaxel (DOCE) vs. other treatments in relation to risks of recurrence of non-muscle-invasive bladder cancer (NMIBC), stratified by study design. Figure S2: Publication bias: funnel plot of studies comparing GEM and/or DOCE to other treatments. Figure S3: Forest plot of studies comparing the efficacy of mitomycin C (MMC) vs. other treatments in relation to risks of recurrence for non-muscle-invasive bladder cancer (NMIBC), stratified by study design. Figure S4: Publication bias: funnel plot of studies comparing MMC to other treatments.

## Abbreviations

Non-muscle-invasive bladder cancer (NMIBC); mitomycin C (MMC); gemcitabine (GEM); docetaxel (DOCE); Bacillus Calmette–Guérin (BCG); repeated transurethral resection of the bladder tumor (TURBT); randomized clinical trial (RCT); retrospective cohort (RC); prospective cohort (PC); relative risk (RR); recurrence-free survival (RFS); confidence interval (CI).
