# Supplementary material for: Comparisons of Intravesical Treatments with Mitomycin C, Gemcitabine, and Docetaxel for Recurrence and Progression of Non-Muscle Invasive Bladder Cancer: Updated Systematic Review and Meta-Analysis
Source: Cancers (Basel). 2024 Dec 10;16(24):4125. doi: 10.3390/cancers16244125 (PMC11674742; doi:10.3390/cancers16244125)

Supplementary Materials

Figure S1: Forest plot of studies comparing the efficacy of gemcitabine (GEM) and/or docetaxel (DOCE) vs. other treatments in relation to risks of recurrence of non-muscle invasive bladder cancer (NMIBC) stratified by study design

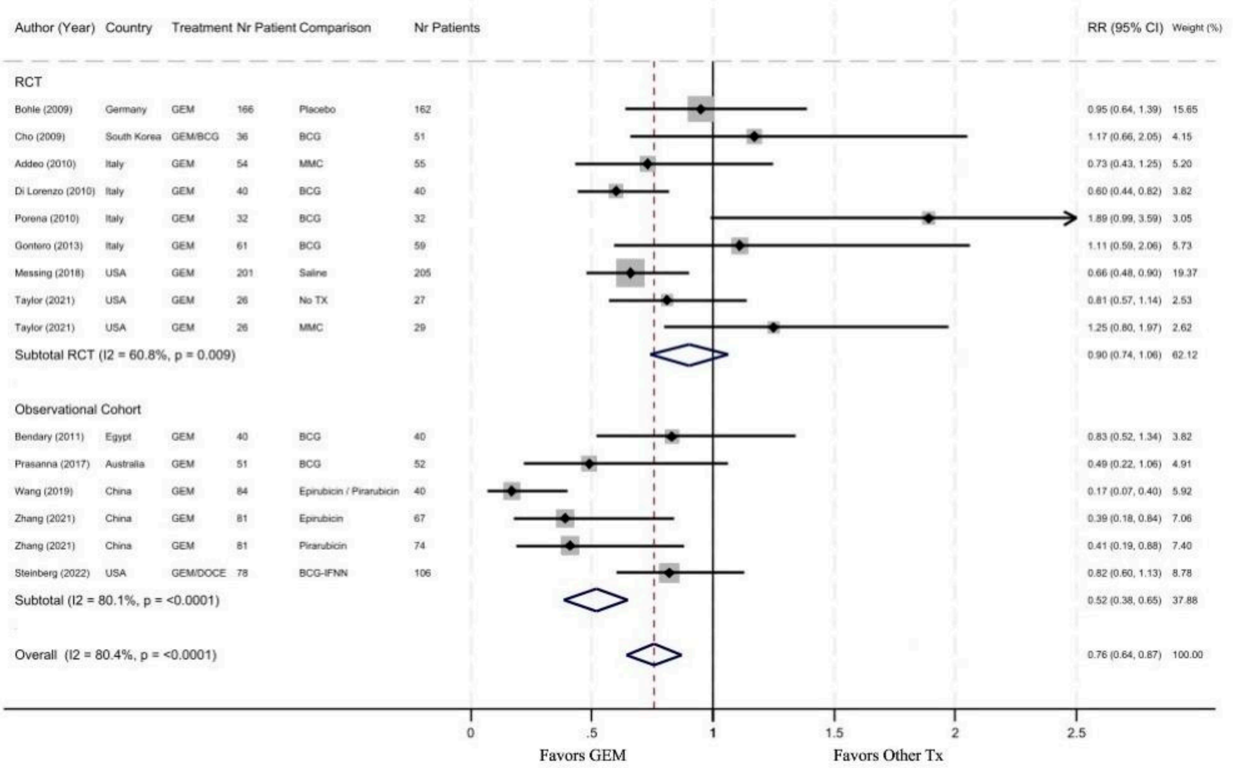

Figure S2: Publication bias: funnel plot of studies comparing GEM and/or DOCE to other treatments

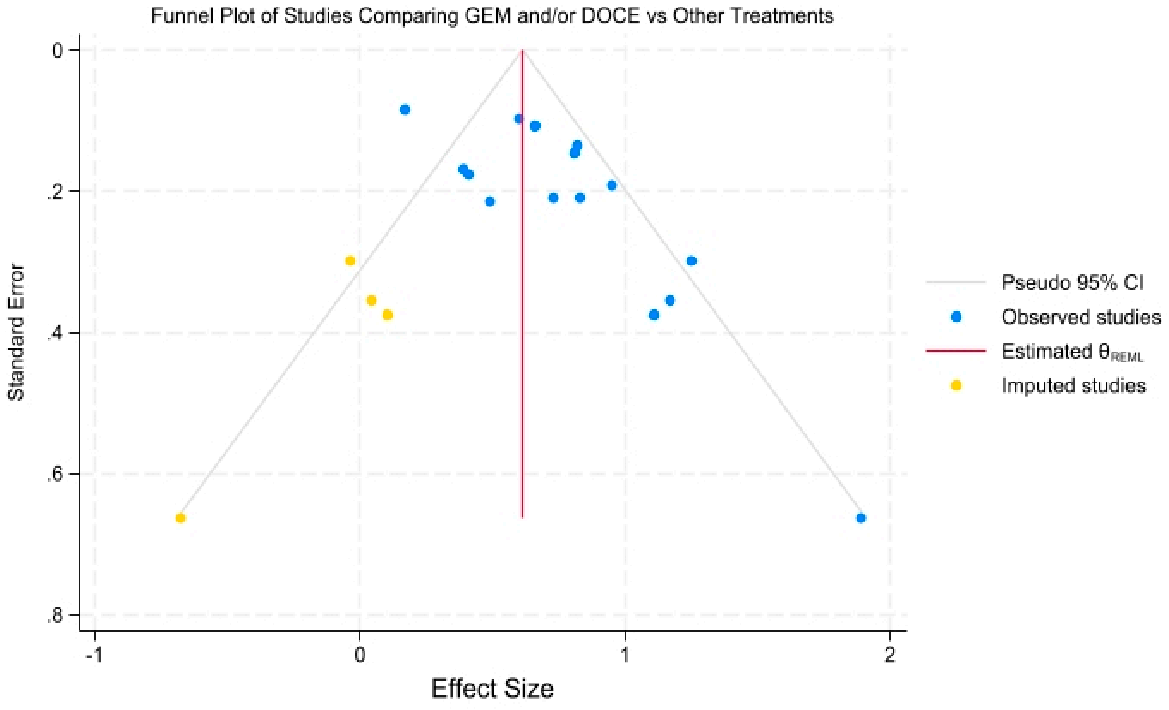

Figure S3: Forest plot of studies comparing the efficacy of mitomycin C (MMC) vs. other treatments in relation to risks of recurrence for non-muscle invasive bladder cancer (NMIBC), stratified by study design

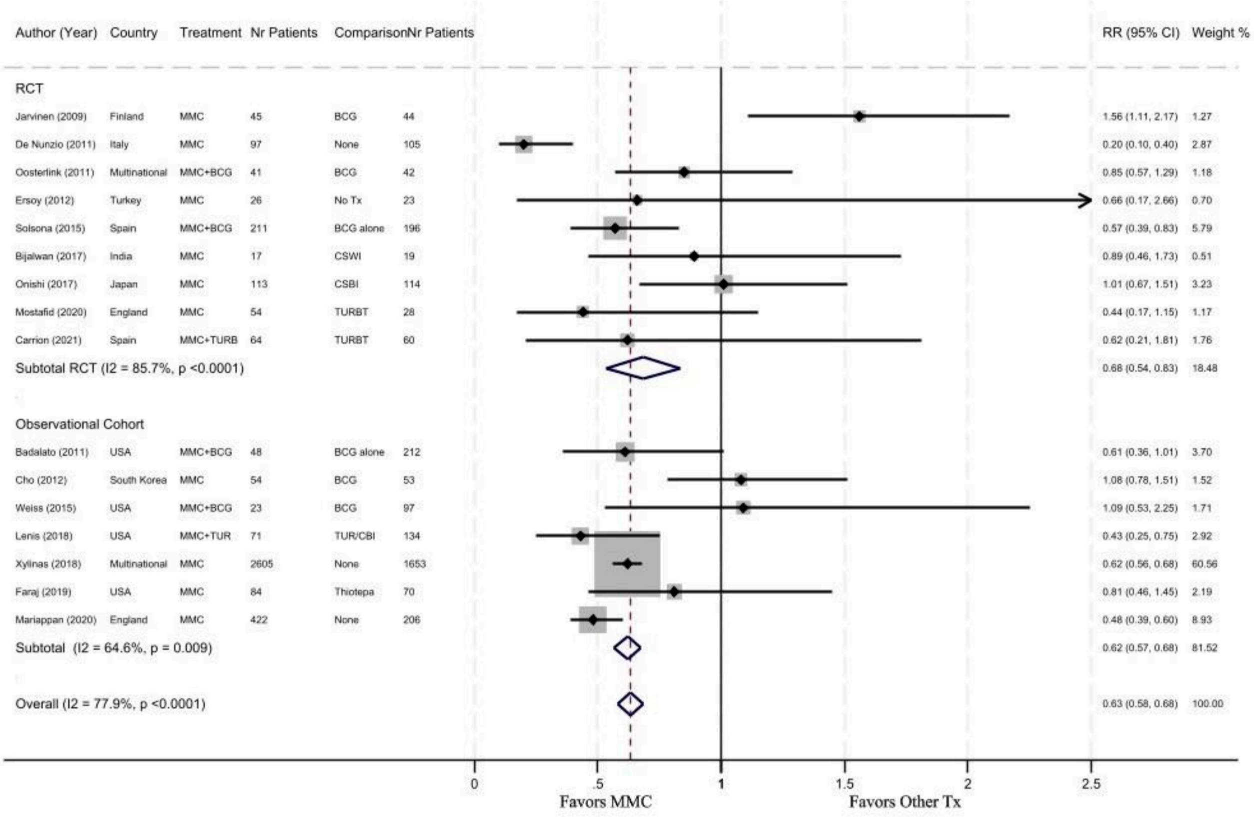

Figure S4: Publication bias: funnel plot of studies comparing MMC to other treatments

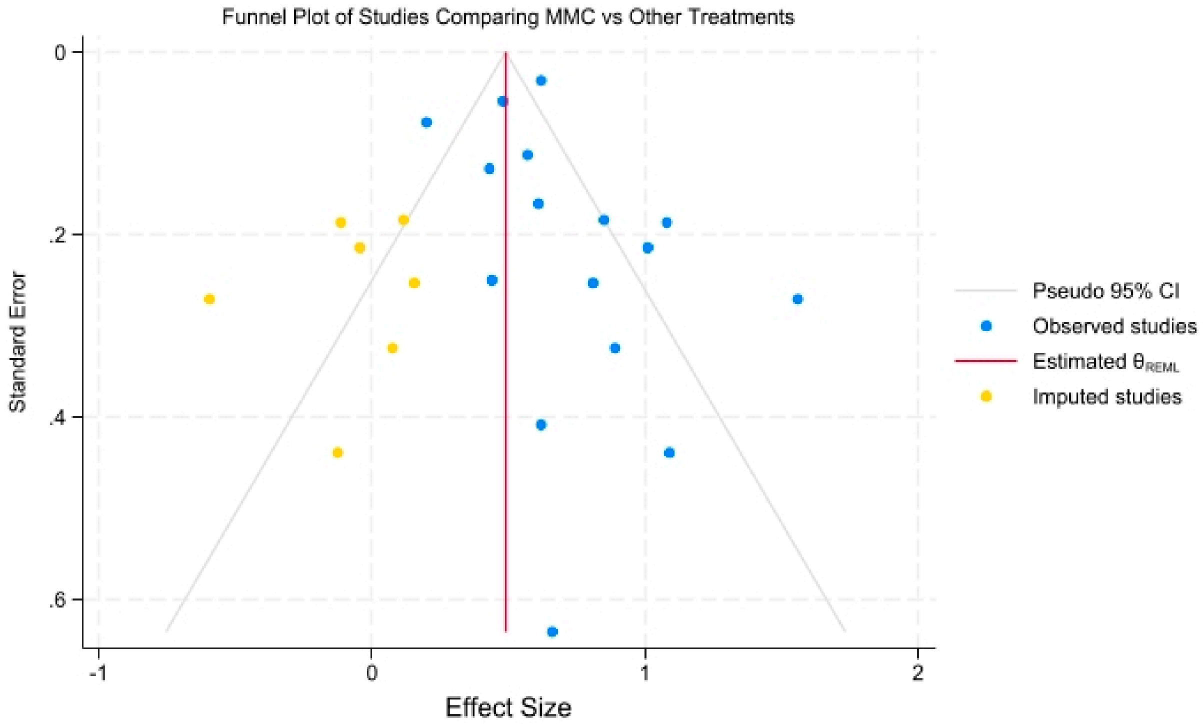

Supplement: Supplementary file 1 [file cancers-16-04125-s001.zip › cancers-3325160-supplementary.pdf]
